# Epidemiological, comorbidity factors with severity and prognosis of COVID-19: a systematic review and meta-analysis

**DOI:** 10.18632/aging.103579

**Published:** 2020-07-13

**Authors:** Xiaoyu Fang, Shen Li, Hao Yu, Penghao Wang, Yao Zhang, Zheng Chen, Yang Li, Liqing Cheng, Wenbin Li, Hong Jia, Xiangyu Ma

**Affiliations:** 1 College of Public Health, Southwest Medical University, Luzhou, Sichuan Province, China; 2 Department of Epidemiology, College of Preventive Medicine, Third Military Medical University, Chongqing, China; 3 The Second Clinical College, Chongqing Medical University, Chongqing, China; 4 Department of Endocrinology, Northern Theater Command General Hospital, Shenyang, China; 5 NCO School of Army Medical University, Shijiazhuang, China; 6 Department of Endocrinology and Metabolism, Southwest Hospital, Third Military Medical University, Chongqing, China; 7 Outpatient Clinic of Dali Retreat Center for Former Cadres of Yunnan Military Region, Dali, China

**Keywords:** COVID-19, meta-analysis, SARS-Cov-2, 2019-nCoV

## Abstract

A systematic review and meta-analysis was conducted in an attempt to systematically collect and evaluate the associations of epidemiological, comorbidity factors with the severity and prognosis of coronavirus disease 2019 (COVID-19). The systematic review and meta-analysis was conducted according to the guidelines proposed by the Preferred Reporting Items for Systematic Reviews and Meta-Analyses (PRISMA). Sixty nine publications met our study criteria, and 61 studies with more than 10,000 COVID-19 cases were eligible for the quantitative synthesis. We found that the males had significantly higher disease severity (RR: 1.20, 95% CI: 1.13-1.27, P <0.001) and more prognostic endpoints. Older age was found to be significantly associated with the disease severity and six prognostic endpoints. Chronic kidney disease contributed mostly for death (RR: 7.10, 95% CI: 3.14-16.02), chronic obstructive pulmonary disease (COPD) for disease severity (RR: 4.20, 95% CI: 2.82-6.25), admission to intensive care unit (ICU) (RR: 5.61, 95% CI: 2.68-11.76), the composite endpoint (RR: 8.52, 95% CI: 4.36-16.65,), invasive ventilation (RR: 6.53, 95% CI: 2.70-15.84), and disease progression (RR: 7.48, 95% CI: 1.60-35.05), cerebrovascular disease for acute respiratory distress syndrome (ARDS) (RR: 3.15, 95% CI: 1.23-8.04), coronary heart disease for cardiac abnormality (RR: 5.37, 95% CI: 1.74-16.54). Our study highlighted that the male gender, older age and comorbidities owned strong epidemiological evidence of associations with the severity and prognosis of COVID-19.

## INTRODUCTION

Since publicly characterized as a pandemic by the World Health Organization on March 11^th^, 2020, the coronavirus disease 2019 (COVID-19) caused by SARS-CoV-2, has raised public concerns globally [1]. As of April 30^th^, 2020, it has caused 3,023,788 confirmed cases and 208,112 deaths [2]. Further, this number is expected to continue to grow rapidly for some time to come, and will threaten the lives, physical and mental health of more people worldwide [3]. To date, there are still no proven specific therapies available for COVID-19, other than supportive cares [4, 5]. It’s a matter of urgency that identifying potential factors affecting the severity and prognosis of COVID-19, and implementing individualized treatment, focused prevention and nursing.

Case-series or retrospective cohort studies have initially explored the associations of epidemiological, comorbidity factors with severity and prognosis of COVID-19 [the multi-stage endpoints including disease severity, acute respiratory distress syndrome (ARDS), an intensive care unit (ICU), the use of mechanical ventilation, or death, etc.] [6–13]. Huang et al. first explored the contribution of demographic and comorbidity factors for ICU admission in 41 COVID-19 cases, and got null results [6]. Further, they conducted a retrospective cohort study with 137 discharged and 54 patients who died, and concluded older age and comorbidities were associated with prognosis of COVID-19 [7]. Meanwhile, in another study with 201 patients, Wu et al. found that older age was associated with higher risk of ARDS and death [8]. A study with 1,590 patients revealed that comorbidities were associated with poorer clinical outcomes [9]. Of note, some studies reported inconsistent, even contradictory conclusions, which might be caused by limited sample size or low endpoint rate [10–13]. Besides, reporting of the same patients in different articles was another concern [14]. Against this context, a thorough understanding of the epidemiological and comorbidity factors upon COVID-19 is urgently warranted. Herein, a systematic review and meta-analysis was conducted to sought to collect and comprehensively evaluate the associations of epidemiological, comorbidity factors with the severity and prognosis of COVID-19.

## RESULTS

### Study characteristics

**Figure 1:**
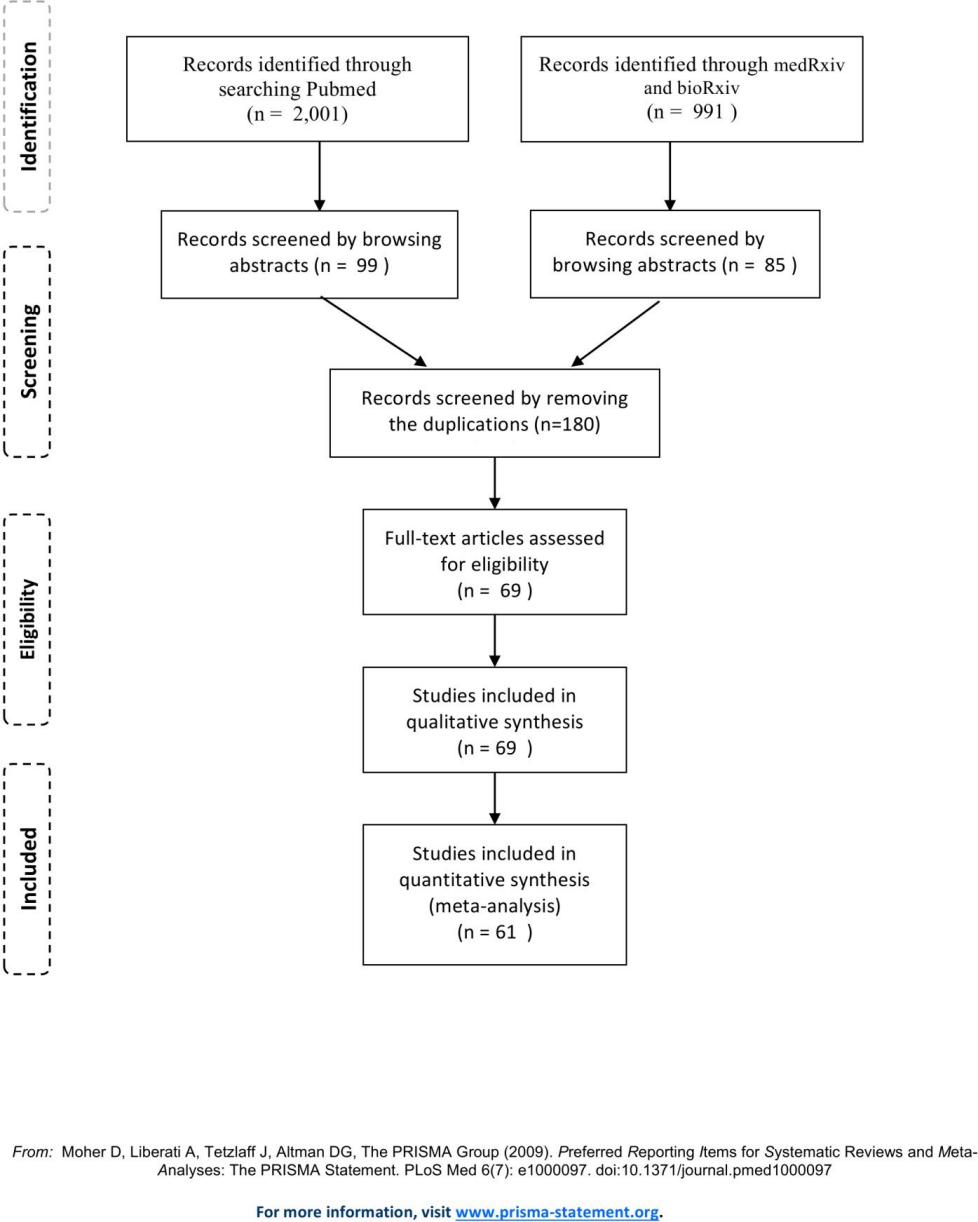
**PRISMA flow diagram.**

Figure 1 presents the PRISMA flow diagram of this study. First, an initial search generated 2,992 potentially relevant papers, of which 2001 identified form Pubmed, and 991 from medRxiv or bioRxiv. After a number of screenings, 69 studies were identified (Supplementary Table 1). Of them, 67 (97.1%) reported Chinese COVID-19 patients, and 2 from either Japan or Singapore. The case number of each study ranged from 21 to 1780, with a mean of 218. The NOS score ranged from 5 to 7, which means a moderate methodological quality. Of the 69 publications, 2 duplicated studies (endpoints, exposure indicators and populations are completely covered by other studies), 4 studies with unique endpoints (survival ≤3d, refractory, liver injury, and time since symptom onset > 10 days), and 2 studies with different grouping methods for disease severity were excluded, which resulted that 61 studies were eventually eligible for the quantitative synthesis (Supplementary Table 1).

### Quantitative data synthesis

The forest plots for all quantitative data synthesis of the epidemiological, comorbidity factors with severity and prognosis of COVID-19 were shown in supplementary materials (Supplementary Figures 1–120). Table 1 presents the quantitative results for the associations of the dichotomous epidemiological, comorbidity factors with severity of COVID-19. First, we found that the males had significant higher disease severity (RR: 1.20, 95% CI: 1.13-1.27, P <0.001, No. of cases: 8916). Besides, comorbidities, including any comorbidities, hypertension, diabetes, malignancy, cardiovascular disease, coronary heart disease, cerebrovascular disease, cardiovascular/cerebrovascular disease, chronic obstructive pulmonary disease (COPD), respiratory system disease, chronic kidney disease, hepatitis B infection, and digestive disease were significantly associated with the disease severity (all P <0.05). Of them, the top 3 effect sizes for the severity of COVID-19 were detected for COPD (RR: 4.20, 95% CI: 2.82-6.25, P<0.001), respiratory system disease (RR: 3.25, 95% CI: 2.48-4.27, P<0.001), and cerebrovascular disease (RR: 2.77, 95% CI: 1.70-4.52, P<0.001).

**Table 1:** Quantitative data synthesis for the associations of the epidemiological, comorbidity factors with severity of COVID-19.

**Variables**	**No of studies**	**Total cases**	**P _heterogeneity_**	**I^2^ (%)**	**RR (95% CIs)**	**P value**	**P _Egger_**
Sex, male	33	8916	0.078	27.2	1.20 (1.13-1.27)	<0.001	0.040
Smoking	11	5237	<0.001	80.8	1.56 (0.95-2.57)	0.082	0.956
Current smoking	2	2879	0.133	55.6	1.17 (0.92-1.50)	0.198	-
Ex-smoking	2	2879	0.019	81.7	2.17 (0.61-7.70)	0.232	-
Drinking	4	2274	0.067	58.0	0.83 (0.48-1.44)	0.516	0.722
Local residents of Wuhan	4	1931	<0.001	90.9	0.66 (0.32-1.36)	0.256	0.441
Exposure to Hubei Province	10	3127	<0.001	90.9	1.21 (0.88-1.65)	0.240	0.115
Contact with confirmed or suspect cases	13	5007	0.041	45.8	0.98 (0.86-1.13)	0.801	0.072
Family cluster	5	2578	0.857	0.0	0.94 (0.86-1.04)	0.224	0.856
Huanan seafood market exposure	5	2342	0.001	79.9	1.79 (0.38-8.35)	0.459	0.212
Comorbidities	16	6219	<0.001	83.4	1.72 (1.44-2.06)	<0.001	0.710
Hypertension	23	7739	<0.001	75.0	2.09 (1.74-2.52)	<0.001	0.154
Diabetes	23	7739	0.017	42.6	1.95 (1.60-2.36)	<0.001	0.272
Malignancy	14	5905	0.137	30.0	1.56 (1.11-2.21)	0.011	0.644
Cardiovascular disease	18	6841	0.019	45.5	2.74 (2.03-3.70)	<0.001	<0.001
Coronary heart disease	8	3899	0.087	43.7	2.03 (1.39-2.15)	<0.001	0.040
Cerebrovascular disease	12	5756	0.074	40.0	2.77 (1.70-4.52)	<0.001	0.595
Cardiovascular/cerebrovascular disease	6	3057	<0.001	84.0	2.31 (1.31-4.08)	0.004	0.502
COPD	14	6609	0.492	0.0	4.20 (2.82-6.25)	<0.001	0.580
Respiratory system disease	18	7522	0.661	0.0	3.25 (2.48-4.27)	<0.001	0.577
Chronic kidney disease	15	4861	0.173	25.5	2.27 (1.55-3.32)	<0.001	0.179
Chronic liver disease	11	3248	0.201	25.5	1.35 (0.89-2.05)	0.165	0.782
Hepatitis B infection	3	1710	0.448	0.0	2.69 (1.32-5.51)	0.007	0.735
Lithiasis	2	308	0.873	0.0	3.03 (0.73-12.58)	0.127	-
Autoimmune disease	5	2202	0.727	0.0	2.52 (0.80-7.90)	0.113	0.997
Abnormal lipid metabolism	4	2246	0.648	0.0	0.57 (0.26-1.25)	0.162	0.080
Digestive disease	5	1013	0.492	0.0	1.80 (1.13-2.87)	0.014	0.717
Thyroid disease	3	348	0.350	0.0	2.37 (0.66-8.50)	0.186	0.387
Tuberculosis	2	592	0.473	0.0	2.74 (0.72-10.4)	0.141	-
Nervous system disease	3	796	0.368	0.0%	1.64 (0.68-3.93)	0.270	0.160
Endocrine system disease	3	796	<0.001	89.6	3.09 (0.70-13.64)	0.136	0.622

We also explored the associations of the dichotomous epidemiological, comorbidity factors with prognosis of COVID-19 (Supplementary Table 2, and Table 2). The males had higher risk of developing the endpoints including death, ARDS, admission to ICU, invasive ventilation, and cardiac abnormality. Hypertension was found to be associated with all seven endpoints, cardiovascular disease and cerebrovascular disease with 6 (except for disease progression), respiratory system disease with 6 (except for Cardiac abnormality), COPD with 5 (except for ARDS and cardiac abnormality), diabetes with 5 (except for the composite endpoint, cardiac abnormality), malignancy with 2 (death, and admission to ICU), etc. Among them, chronic kidney disease contributed mostly for death (RR: 7.10, 95% CI: 3.14-16.02, P<0.001), COPD for admission to ICU (RR: 5.61, 95% CI: 2.68-11.76, P<0.001), the composite endpoint (RR: 8.52, 95% CI: 4.36-16.65, P<0.001), invasive ventilation (RR: 6.53, 95% CI: 2.70-15.84, P<0.001), and disease progression (RR: 7.48, 95% CI: 1.60-35.05, P =0.011), cerebrovascular disease for ARDS (RR: 3.15, 95% CI: 1.23-8.04, P =0.016), coronary heart disease for cardiac abnormality (RR: 5.37, 95% CI: 1.74-16.54, P =0.003). Besides, the associations of continuous age with severity and prognosis of COVID-19 were presented in Table 3. Older age was found to be significantly associated with the disease severity and six endpoints (all P value <0.001, except a marginal association for disease progression). The biggest standard mean difference (SMD) was detected for death (SMD: 1.06, 95% CI: 0.85-1.26, P<0.001). However, we didn’t find any statistically significant associations for epidemiological factors, including drinking, local residents of Wuhan, exposure to Hubei Province, contact with confirmed or suspect cases, family cluster, and Huanan seafood market exposure. Sensitivity analyses by changing the pooling model and statistical variables, or using one-at-a-time method, were performed to assess the stability of the results. However, we found the results were not materially changed (data not shown). Further, we applied the Egger test to evaluated the potential publication bias, and very litter evidence (among all 120 associations, only 6 presented the existence of possible publication bias) was detected (Tables 1–3 and Supplementary Table 2).

**Table 2:** Quantitative data synthesis for the associations of the epidemiological, comorbidity factors with prognosis of COVID-19 (P value<0.05).

**Variables**	**No of studies**	**Total cases**	**P _heterogeneity_**	**I^2^ (%)**	**RR (95% CIs)**	**P value**	**P _Egger_**
Death							
Sex, male	10	4214	0.443	0.0	1.23 (1.14-1.33)	<0.001	0.276
Comorbidities	8	4499	<0.001	88.7	1.68 (1.32-2.13)	<0.001	0.248
Hypertension	11	4860	<0.001	84.4	1.74 (1.31-2.30)	<0.001	0.418
Diabetes	10	4748	0.001	67.1	1.75 (1.27-2.41)	0.001	0.057
Malignancy	6	3978	0.262	22.8	3.09 (1.59-6.00)	0.001	0.006
Cardiovascular disease	11	4860	<0.001	75.9	2.67 (1.60-4.43)	<0.001	0.654
Coronary heart disease	5	2452	<0.001	87.7	3.16 (1.45-6.91)	0.004	0.435
Cerebrovascular disease	6	3771	0.457	0.0	4.61 (2.51-8.47)	<0.001	0.766
COPD	4	3677	0.279	22.0	5.31 (2.63-10.71)	<0.001	0.107
Respiratory system disease	7	4472	0.185	31.8	3.22 (2.12-4.90)	<0.001	0.761
Chronic kidney disease	5	2219	0.477	0.0	7.10 (3.14-16.02)	<0.001	0.772
Admission to ICU							
Sex, male	5	2224	0.011	69.6	1.29 (1.13-1.47)	<0.001	0.651
Comorbidities	5	3747	0.038	60.5	1.82 (1.45-2.29)	<0.001	0.646
Hypertension	5	3747	0.601	0.0	2.31 (1.97-2.70)	<0.001	0.312
Diabetes	5	3747	0.084	51.4	1.88 (1.10-3.23)	0.021	0.457
Malignancy	5	3747	0.427	0.0	2.52 (1.38-5.59)	0.003	0.158
Cardiovascular disease	5	3747	0.511	0.0	2.74 (1.92-3.92)	<0.001	0.692
Cerebrovascular disease	3	3508	0.349	4.9	5.12 (2.86-9.17)	<0.001	0.273
COPD	4	3549	0.800	0.0	5.61 (2.68-11.76)	<0.001	0.740
Respiratory system disease	4	3549	0.613	0.0	4.66 (2.59-8.40)	<0.001	0.637
Composite endpoint							
Smoking	2	2879	0.604	0.0	2.67 (1.91-3.73)	<0.001	-
Comorbidities	2	3370	<0.001	95.3	1.96 (1.06-3.60)	0.031	-
Hypertension	2	3370	0.011	84.5	2.20 (1.44-3.36)	<0.001	-
Cardiovascular disease	2	3370	0.927	0.0	3.09 (2.09-4.57)	<0.001	-
Coronary heart disease	2	3370	0.473	0.0	3.36 (2.15-5.25)	<0.001	-
Cerebrovascular disease	2	3370	0.225	32.0	4.10 (2.34-7.18)	<0.001	-
COPD	2	3370	0.185	43.0	8.52 (4.36-16.65)	<0.001	-
Respiratory system disease	2	3370	0.185	43.0	8.52 (4.36-16.65)	<0.001	-
ARDS							
Sex, male	3	2090	0.464	0.0	1.15 (1.01-1.30)	0.033	0.353
Hypertension	3	2090	0.377	0.0	1.90 (1.57-2.30)	<0.001	0.520
Diabetes	3	2090	0.068	62.9	3.07 (1.28-7.36)	0.012	0.066
Cardiovascular disease	3	2090	0.244	29.2	2.26 (1.43-3.58)	<0.001	0.422
Cerebrovascular disease	2	1889	0.152	51.2	3.15 (1.23-8.04)	0.016	-
Respiratory system disease	2	1889	0.303	5.6	2.44 (1.20-4.97)	0.014	-
Invasive ventilation							
Sex, male	2	1825	0.403	0.0	1.35 (1.11-1.64)	0.002	-
Family cluster	2	1825	0.646	0.0	1.58 (1.13-2.14)	0.006	-
Comorbidities	3	3415	0.005	81.2	1.83 (1.19-2.79)	0.006	0.569
Hypertension	3	3415	0.131	50.9	2.35 (1.92-2.89)	<0.001	0.366
Diabetes	3	3415	0.131	50.8	1.85 (1.24-2.76)	0.003	0.021
Cardiovascular disease	3	3415	0.844	0.0	2.90 (1.63-5.15)	<0.001	0.618
Cerebrovascular disease	2	3370	0.602	0.0	3.98 (1.77-8.93)	0.001	-
COPD	2	3370	0.383	0.0	6.53 (2.70-15.84)	<0.001	-
Respiratory system disease	3	3415	0.260	25.7	4.34 (2.04-9.26)	<0.001	0.567
Cardiac abnormality							
Sex, male	4	439	0.211	33.6	1.33 (1.02-1.72)	0.036	0.624
Hypertension	4	439	0.947	0.0	2.97 (1.65-5.34)	<0.001	0.610
Cardiovascular disease	4	439	0.915	0.0	4.90 (1.82-13.21)	0.002	0.177
Coronary heart disease	3	386	0.819	0.0	5.37 (1.74-16.54)	0.003	0.408
Disease progression							
Hypertension	2	219	0.547	0.0	2.90 (1.45-5.81)	0.003	-
Diabetes	2	219	0.746	0.0	3.30 (1.08-10.07)	0.036	-
COPD	2	219	0.848	0.0	7.48 (1.60-35.05)	0.011	-
Respiratory system disease	2	219	0.848	0.0	7.48 (1.60-35.05)	0.011	-

**Table 3:** Quantitative data synthesis for the associations of age with severity and prognosis of COVID-19.

**Variables**	**No of studies**	**Total cases**	**P _heterogeneity_**	**I^2^ (%)**	**SMD (95% CIs)**	**P value**	**P _Egger_**
Severity	32	8140	<0.001	92.4	0.73 (0.53-0.94)	<0.001	0.331
Death	9	3725	0.005	63.9	1.06 (0.85-1.26)	<0.001	0.610
Admission to ICU	5	2224	0.189	34.9	0.78 (0.60-0.96)	<0.001	0.538
Composite endpoint	2	2879	0.055	72.9	0.88 (0.56-1.21)	<0.001	-
ARDS	3	2090	0.939	0	0.83 (0.67-0.99)	<0.001	0.882
Invasive ventilation	2	1825	0.493	0.0	0.84 (0.54-1.14)	<0.001	-
Cardiac abnormality	4	439	0.041	63.6	0.92 (0.44-1.41)	<0.001	0.885
Disease progression	2	219	<0.001	95.4	2.37 (0.00-4.74)	0.050	-

## DISCUSSION

To our knowledge, this should be the most comprehensive assessment of epidemiological, comorbidity factors with the severity and prognosis of COVID-19 conducted to date. We systematically evaluated data for more than ten thousand COVID-19 cases from 69 publications in the past several months, and identified that the males had higher risk of reaching severe disease and adverse prognostic endpoints. Older age was found to be significantly associated with the disease severity and six prognostic endpoints. Comorbidities, including hypertension, diabetes, cardiovascular disease, cerebrovascular disease, COPD, chronic kidney disease, and malignancy, contributed significantly to the disease severity and prognostic endpoints of COVID-19. Results from the current study would be helpful for implementing individualized treatment, focused prevention and nursing of COVID-19.

The “Gender and COVID-19 Working Group” first raised the concern of the gendered impacts of the COVID-19 outbreak [15], then echoed by another two publications [16, 17]. In a large epidemiological investigation in China with 72,314 cases, 51.0% of the patients were the males [18]. In a recent report of 1,590 hospitalized COVID-19 patients in China [9], the male rate was 57.3%, and it was 51.3% in Huoshenshan study with 1780 hospitalized cases. All these evidence indicated the almost equal sex distribution and disease susceptibility. Despite this, we identified that the males had higher rate of severity and prognostic endpoints in our meta-analysis. This finding was indirectly proved by a Italian study with 1591 ICU patients, the male rate of which was 82.0%, and higher than that previously reported [19]. However, the smoking status which has significant gender predisposition, showed no statistical associations with disease severity and prognosis of COVID-19, except for composite endpoint. The relationship between smoking and COVID-19 has become a very controversial topic, and should be interpreted with caution, as many factors could affect the results, such as the statistical power, definition of smoking status, the presence of confounding factors, and the potential role of angiotensin-converting enzyme-2 (ACE-2) [20–22]. Older age was another strong determinant of disease referral and outcomes in our results, which has been proved by a model-based analysis [23], and supported by studies of severe acute respiratory syndrome (SARS) and middle east respiratory syndrome (MERS) [24, 25].

In addition to epidemiological factors, comorbidities are also potentially important aspects which could affect the disease severity and prognosis of COVID-19. As a key regulator of blood pressure, angiotensin-converting enzyme (ACE) was also the binding site of SARS-CoV, making hypertension the most focused comorbidity [26, 27]. In our meta-analysis, we found hypertension was associated with higher rate of the disease severity and all prognostic endpoints. Of note, using of angiotensin-converting enzyme inhibitors (ACEIs) and angiotensin II type 1 receptor blockers (ARBs) could contribute to the improvement of outcomes of COVID-19 patients with hypertension [28]. COPD was a major predominant indicator for the disease severity and prognosis of COVID-19. In our study, COPD contributed most to the admission to ICU, the composite endpoint, and invasive ventilation of COVID-19. Among the comorbidities, the contribution of malignancy to the prognosis of COVID-19 was a controversial topic. Liang et al. [29] first reported that patients with cancer had a higher risk of COVID-19 and with a poorer prognosis than those without cancer, then challenged by two other publications because of the sample size, and confounding factors [30, 31]. Our meta-analysis temporarily supported Liang’s conclusion that malignancy contributed to death, and admission to ICU with a moderate sample size, although we can’t adjusted for the potential confounding bias. An interesting finding was that chronic kidney disease contributed mostly to the death. It is likely an immunologic explanation, given our current understanding of weakened immune system in patients with chronic kidney disease [32]. A more targeted and intensive health protection strategy for the patients with comorbidities above may be warranted.

The strengths of our study included an extensive systematic search strategy, a thorough examination of duplicate data, and a comprehensive quality assessment of the primary studies. The findings in the current study are also affected by several limitations. First, although we have systematically searched the literature to identify eligible studies, it is possible that some studies might have been missed. Despite the wide ranging search strategy, non-English language studies might not have been indexed in the databases we searched. Second, all studies except for two studies from Japan or Singapore, were from China in the early stage of the COVID-19 outbreak. This limited the findings’ applicability across different populations and geographic regions during the COVID-19 pandemic. Third, although we have screened the hospital name, date of recruitment aiming to find duplicated usage of cases, it is inevitable as some studies used samples from multiple hospitals and have not reported detailed patient composition. This might affect the accurate estimates of disease prevalence or outcomes of COVID-19. Fourth, significant study heterogeneity or publication bias which may lead to questionable interpretation of result were detected for some associations (especially for comorbidities). Thus, these results should be interpreted with caution. Finally, moderate NOS score means the flawed methodological quality. However, as these studies were dealing with urgent public health concerns and carried out in a state of emergency, the quality was within acceptable limits. Taken together, despite some limitations, our study provides an important basis for a comprehensive understanding of disease severity and prognosis-related factors.

## CONCLUSIONS

In our systematic review and meta-analysis, we highlighted that the male gender, older age and comorbidities showed strong epidemiological evidence of associations with the severity and prognosis COVID-19. Taken together, this large-scale meta-analysis not only summarizes the current literatures upon associations of epidemiological, comorbidity factors with the severity and prognosis of COVID-19, but also provides helpful clues for implementing individualized treatment, focused prevention and nursing of COVID-19. Further well-designed prospective cohort studies and randomized controlled trials are warranted to explore the severity and prognosis related factors of COVID-19.

## MATERIALS AND METHODS

### Search strategy and selection criteria

The systematic review and meta-analyses were conducted and reported according to the guidelines proposed by the PRISMA [33]. Studies were eligible for inclusion in this meta-analysis if they met the following criteria: (1) data published in a peer-reviewed journal in English or Chinese; (2) the study is a case-control, cohort, or a cross-sectional design in human beings; (3) the studies provide sufficient information for epidemiological, comorbidity factors with severity or prognosis of COVID-19; (4) When multiple publications reported on the same hospital, date of recruitment, exposures and endpoints, we defined it as duplicated studies. Some studies, although duplicate in terms of hospital and date of recruitment, were not judged to be duplicate because they evaluated different exposure indicators or endpoints.

Literature retrieval was conducted through a two-step strategy with a cut-off date of April 5^th^, 2020 (Figure 1). In step 1, we searched the PubMed database using the following key terms in combination: “2019-nCoV OR COVID-19 OR covid-2019 OR novel Coronavirus–Infected Pneumonia OR novel coronavirus OR SARS-CoV-2 OR Wuhan Coronavirus OR Wuhan pneumonia”. In step 2, the COVID-19 or SARS-CoV-2 preprints were also retrieved from the medRxiv (https://www.medrxiv.org/) and bioRxiv (https://www.biorxiv.org/) databases using the above terms sequentially. We also searched the references and related articles of all gathered papers, and checked previously published meta-analyses and reviews. In the current study, we also incorporated the data of 1780 COVID-19 cases from Wuhan Huoshenshan hospital, the first and largest emergency specialty field hospital in epicenter Wuhan, China. Finally, 69 publications met our study criteria were included in the systematic review, and 61 studies were eligible for the quantitative synthesis.

### Data extraction and quality control

All data were extracted by at least two authors (FX, LS, YH, and WP) according to the pre-specified selection criteria. Disagreement was resolved by discussion with a third party personnel. The details for each study including the first author, country, city or province, year of publication, source hospitals, date duration of the patient recruitment, PubMed identifier number (PMID) or the digital object identifier (DOI) number, total sample size, disease severity, clinical endpoints, the distribution of epidemiological and comorbidity factors, were extracted using a structured data sheet. Frequency numbers of dichotomous variables, and median (IQR, interquartile range) or mean (SD, standard deviation) for continuous variables were recorded. Median (IQR) were transferred to the form of mean (SD) using the method recommended by the Cochrane handbook version 6, 2019. The endpoints consisted of disease severity, ARDS, admission to ICU, death, a composite endpoint, invasive ventilation, cardiac abnormality, and disease progression (the detailed definitions of the endpoints were presented in supplementary methods). The degree of severity of COVID-19 was determined using the American Thoracic Society guidelines for community-acquired pneumonia or the New Coronavirus Pneumonia Prevention and Control Guidelines of China [34, 35]. Two authors independently evaluated the methodological quality of included studies using the Newcastle-Ottawa Scale (NOS) for cohort study [36]. Any disagreement in the quality assessment was resolved by discussion with a third author. The NOS includes 8 items (up to 9 stars), each one of these items was scored from 0 to 1, except that a maximum of two stars can be given for comparability.

### Data synthesis

All statistical analyses for this study were performed by STATA, version 12.0 (Stata Corporation, College Station, Texas). All tests were two-sided, and a *P*-value of less than 0.05 for any test or model was considered statistically significant unless otherwise stated. Meta-analysis was performed for all associations with data available from 2 or more independent samples. Summary relative ratios (RRs), or standard mean difference (SMD) with their 95% confidence intervals (CIs) were used to assess the strength of associations between epidemiological, comorbidity factors with the severity and prognosis of COVID-19 by either the fixed-effect model (Mantel–Haenszel method) or, in case of heterogeneity, the random-effect model (DerSimonian–Laird method). To assess inter-study heterogeneity, we calculated the chi-square-based Cochran’s *Q* statistic test and I^2^ statistic. Because of the low power of Cochran’s *Q* statistic, heterogeneity was considered significant if *P* < 0.10. For I^2^, values around 25% indicated low heterogeneity, around 50% moderate heterogeneity, and around 75% high heterogeneity. Publication bias was assessed visually by funnel plots and quantitatively with Egger’ s regression test for asymmetry.

## Supplementary Material

Supplementary Methods, Figures and Tables
