# Supplementary material for: Epidemiological, comorbidity factors with severity and prognosis of COVID-19: a systematic review and meta-analysis
Source: Aging (Albany NY). 2020 Jul 13;12(13):12493–503. doi: 10.18632/aging.103579 (PMC7377860; doi:10.18632/aging.103579)
Supplement: Supplementary Methods, Figures and Tables [file aging-12-103579-s001..docx]

Supplementary materials

**Table of Contents**

[Supplementary methods 6](#_Toc37230252)

[The Detailed Definitions of the Severity and Prognostic Endpoints of COVID-19 6](#_Toc37230253)

[Table S1 Characteristics of the included studies. 8](#_Toc37230254)

[Table S2 Quantitative data synthesis for the associations of the epidemiological, comorbidity factors with prognosis of COVID-19 16](#_Toc37230255)

[Figure S1 Forest plot of association between sex and disease severity. 20](#_Toc37230256)

[Figure S2 Forest plot of association between smoking and disease severity. 21](#_Toc37230257)

[Figure S3 Forest plot of association between current smoker and disease severity. 22](#_Toc37230258)

[Figure S4 Forest plot of association between ex-smoker and disease severity. 23](#_Toc37230259)

[Figure S5 Forest plot of association between drinking and disease severity. 24](#_Toc37230260)

[Figure S6 Forest plot of association between local residents of Wuhan and disease severity. 25](#_Toc37230261)

[Figure S7 Forest plot of association between exposure history to Hubei province and disease severity. 26](#_Toc37230262)

[Figure S8 Forest plot of association between contact with confirmed or suspect cases and disease severity. 27](#_Toc37230263)

[Figure S9 Forest plot of association between family cluster and disease severity. 28](#_Toc37230264)

[Figure S10 Forest plot of association between Huanan seafood market exposure and disease severity. 29](#_Toc37230265)

[Figure S11 Forest plot of association between comorbidity and disease severity. 30](#_Toc37230266)

[Figure S12 Forest plot of association between hypertension and disease severity. 31](#_Toc37230267)

[Figure S13 Forest plot of association between diabetes and disease severity. 32](#_Toc37230268)

[Figure S14 Forest plot of association between malignancy and disease severity. 33](#_Toc37230269)

[Figure S15 Forest plot of association between cardiovascular disease and disease severity. 34](#_Toc37230270)

[Figure S16 Forest plot of association between coronary heart disease and disease severity. 35](#_Toc37230271)

[Figure S17 Forest plot of association between cerebrovascular disease and disease severity. 36](#_Toc37230272)

[Figure S18 Forest plot of association between cardiovascular/ cerebrovascular disease and disease severity. 37](#_Toc37230273)

[Figure S19 Forest plot of association between COPD and disease severity. 38](#_Toc37230274)

[Figure S20 Forest plot of association between respiratory system disease and disease severity. 39](#_Toc37230275)

[Figure S21 Forest plot of association between chronic kidney disease and disease severity. 40](#_Toc37230276)

[Figure S22 Forest plot of association between chronic liver disease and disease severity. 41](#_Toc37230277)

[Figure S23 Forest plot of association between hepatitis B infection and disease severity. 42](#_Toc37230278)

[Figure S24 Forest plot of association between lithiasis and disease severity. 43](#_Toc37230279)

[Figure S25 Forest plot of association between autoimmune disease and disease severity. 44](#_Toc37230280)

[Figure S26 Forest plot of association between abnormal lipid metabolism and disease severity. 45](#_Toc37230281)

[Figure S27 Forest plot of association between digestive disease and disease severity. 46](#_Toc37230282)

[Figure S28 Forest plot of association between thyroid disease and disease severity. 47](#_Toc37230283)

[Figure S29 Forest plot of association between tuberculosis and disease severity. 48](#_Toc37230284)

[Figure S30 Forest plot of association between nervous system disease and disease severity. 49](#_Toc37230285)

[Figure S31 Forest plot of association between endocrine system disease and disease severity. 50](#_Toc37230286)

[Figure S32 Forest plot of association between death and sex. 51](#_Toc37230287)

[Figure S33 Forest plot of association between death and smoking. 52](#_Toc37230288)

[Figure S34 Forest plot of association between death and current smoking. 53](#_Toc37230289)

[Figure S35 Forest plot of association between death and ex-smoking. 54](#_Toc37230290)

[Figure S36 Forest plot of association between death and contact with confirmed or suspect cases. 55](#_Toc37230291)

[Figure S37 Forest plot of association between death and Huanan seafood market exposure. 56](#_Toc37230292)

[Figure S38 Forest plot of association between death and comorbidities. 57](#_Toc37230293)

[Figure S39 Forest plot of association between death and hypertension. 58](#_Toc37230294)

[Figure S40 Forest plot of association between death and diabetes. 59](#_Toc37230295)

[Figure S41 Forest plot of association between death and malignancy. 60](#_Toc37230296)

[Figure S42 Forest plot of association between death and cardiovascular disease. 61](#_Toc37230297)

[Figure S43 Forest plot of association between death and coronary heart disease. 62](#_Toc37230298)

[Figure S44 Forest plot of association between death and cerebrovascular disease. 63](#_Toc37230299)

[Figure S45 Forest plot of association between death and COPD. 64](#_Toc37230300)

[Figure S46 Forest plot of association between death and respiratory system disease. 65](#_Toc37230301)

[Figure S47 Forest plot of association between death and chronic kidney disease. 66](#_Toc37230302)

[Figure S48 Forest plot of association between death and hepatitis B infection. 67](#_Toc37230303)

[Figure S49 Forest plot of association between death and autoimmune disease. 68](#_Toc37230304)

[Figure S50 Forest plot of association between admission to ICU and sex. 69](#_Toc37230305)

[Figure S51 Forest plot of association between admission to ICU and smoking. 70](#_Toc37230306)

[Figure S52 Forest plot of association between admission to ICU and drinking. 71](#_Toc37230307)

[Figure S53 Forest plot of association between admission to ICU and Huanan seafood market exposure. 72](#_Toc37230308)

[Figure S54 Forest plot of association between admission to ICU and comorbidities. 73](#_Toc37230309)

[Figure S55 Forest plot of association between admission to ICU and hypertension. 74](#_Toc37230310)

[Figure S56 Forest plot of association between admission to ICU and diabetes. 75](#_Toc37230311)

[Figure S57 Forest plot of association between admission to ICU and malignancy. 76](#_Toc37230312)

[Figure S58 Forest plot of association between admission to ICU and cardiovascular disease. 77](#_Toc37230313)

[Figure S59 Forest plot of association between admission to ICU and cerebrovascular disease. 78](#_Toc37230314)

[Figure S60 Forest plot of association between admission to ICU and COPD. 79](#_Toc37230315)

[Figure S61 Forest plot of association between admission to ICU and respiratory system disease. 80](#_Toc37230316)

[Figure S62 Forest plot of association between admission to ICU and chronic kidney disease. 81](#_Toc37230317)

[Figure S63 Forest plot of association between admission to ICU and chronic liver disease. 82](#_Toc37230318)

[Figure S64 Forest plot of association between composite endpoint and sex. 83](#_Toc37230319)

[Figure S65 Forest plot of association between composite endpoint and smoking. 84](#_Toc37230320)

[Figure S66 Forest plot of association between composite endpoint and current smoking. 85](#_Toc37230321)

[Figure S67 Forest plot of association between composite endpoint and ex-smoking. 86](#_Toc37230322)

[Figure S68 Forest plot of association between composite endpoint and contact with confirmed or suspect cases. 87](#_Toc37230323)

[Figure S69 Forest plot of association between composite endpoint and comorbidities. 88](#_Toc37230324)

[Figure S70 Forest plot of association between composite endpoint and hypertension. 89](#_Toc37230325)

[Figure S71 Forest plot of association between composite endpoint and diabetes. 90](#_Toc37230326)

[Figure S72 Forest plot of association between composite endpoint and malignancy. 91](#_Toc37230327)

[Figure S73 Forest plot of association between composite endpoint and cardiovascular disease. 92](#_Toc37230328)

[Figure S74 Forest plot of association between composite endpoint and coronary heart disease. 93](#_Toc37230329)

[Figure S75 Forest plot of association between composite endpoint and cerebrovascular disease. 94](#_Toc37230330)

[Figure S76 Forest plot of association between composite endpoint and COPD. 95](#_Toc37230331)

[Figure S77 Forest plot of association between composite endpoint and respiratory system disease. 96](#_Toc37230332)

[Figure S78 Forest plot of association between ARDS and sex. 97](#_Toc37230333)

[Figure S79 Forest plot of association between ARDS and hypertension. 98](#_Toc37230334)

[Figure S80 Forest plot of association between ARDS and diabetes. 99](#_Toc37230335)

[Figure S81 Forest plot of association between ARDS and cardiovascular disease. 100](#_Toc37230336)

[Figure S82 Forest plot of association between ARDS and cerebrovascular disease. 101](#_Toc37230337)

[Figure S83 Forest plot of association between ARDS and COPD. 102](#_Toc37230338)

[Figure S84 Forest plot of association between ARDS and respiratory system disease. 103](#_Toc37230339)

[Figure S85 Forest plot of association between invasive ventilation and sex. 104](#_Toc37230340)

[Figure S86 Forest plot of association between invasive ventilation and smoking. 105](#_Toc37230341)

[Figure S87 Forest plot of association between invasive ventilation and contact with confirmed or suspect cases. 106](#_Toc37230342)

[Figure S88 Forest plot of association between invasive ventilation and family cluster. 107](#_Toc37230343)

[Figure S89 Forest plot of association between invasive ventilation and comorbidities. 108](#_Toc37230344)

[Figure S90 Forest plot of association between invasive ventilation and hypertension. 109](#_Toc37230345)

[Figure S91 Forest plot of association between invasive ventilation and diabetes. 110](#_Toc37230346)

[Figure S92 Forest plot of association between invasive ventilation and malignancy. 111](#_Toc37230347)

[Figure S93 Forest plot of association between invasive ventilation and cardiovascular disease. 112](#_Toc37230348)

[Figure S94 Forest plot of association between invasive ventilation and cerebrovascular disease. 113](#_Toc37230349)

[Figure S95 Forest plot of association between invasive ventilation and COPD. 114](#_Toc37230350)

[Figure S96 Forest plot of association between invasive ventilation and respiratory system disease. 115](#_Toc37230351)

[Figure S97 Forest plot of association between cardiac abnormality and sex. 116](#_Toc37230352)

[Figure S98 Forest plot of association between cardiac abnormality and smoking. 117](#_Toc37230353)

[Figure S99 Forest plot of association between cardiac abnormality and exposure to Hubei Province. 118](#_Toc37230354)

[Figure S100 Forest plot of association between cardiac abnormality and contact with confirmed or suspect cases. 119](#_Toc37230355)

[Figure S101 Forest plot of association between cardiac abnormality and hypertension. 120](#_Toc37230356)

[Figure S102 Forest plot of association between cardiac abnormality and diabetes. 121](#_Toc37230357)

[Figure S103 Forest plot of association between cardiac abnormality and cardiovascular disease. 122](#_Toc37230358)

[Figure S104 Forest plot of association between cardiac abnormality and coronary heart disease. 123](#_Toc37230359)

[Figure S105 Forest plot of association between cardiac abnormality and COPD. 124](#_Toc37230360)

[Figure S106 Forest plot of association between cardiac abnormality and respiratory system disease. 125](#_Toc37230361)

[Figure S107 Forest plot of association between disease progression and sex. 126](#_Toc37230362)

[Figure S108 Forest plot of association between disease progression and smoking. 127](#_Toc37230363)

[Figure S109 Forest plot of association between disease progression and hypertension. 128](#_Toc37230364)

[Figure S110 Forest plot of association between disease progression and diabetes. 129](#_Toc37230365)

[Figure S111 Forest plot of association between disease progression and COPD. 130](#_Toc37230366)

[Figure S112 Forest plot of association between disease progression and respiratory system disease. 131](#_Toc37230367)

[Figure S113 Forest plot of association between age and severity. 132](#_Toc37230368)

[Figure S114 Forest plot of association between age and death. 133](#_Toc37230369)

[Figure S115 Forest plot of association between age and admission to ICU. 134](#_Toc37230370)

[Figure S116 Forest plot of association between age and composite endpoint. 135](#_Toc37230371)

[Figure S117 Forest plot of association between age and ARDS. 136](#_Toc37230372)

[Figure S118 Forest plot of association between age and invasive ventilation. 137](#_Toc37230373)

[Figure S119 Forest plot of association between age and cardiac abnormality. 138](#_Toc37230374)

[Figure S120 Forest plot of association between age and disease progression. 139](#_Toc37230375)

Supplementary methods

# **The Detailed Definitions of the** Severity and **Prognostic Endpoints of** COVID-19

1. The degree of severity of COVID-19 was determined using the American Thoracic Society guidelines for community-acquired pneumonia or the New Coronavirus Pneumonia Prevention and Control Guidelines of China [1, 2]. The latter grouped COVID-19 patients into four categories: (1) mild type: patients with mild clinical symptoms and no pulmonary changes on CT imaging; (2) common type: patients with symptoms of fever and signs of respiratory infection, and having pneumonia changes on CT imaging; (3) severe type: patients presenting with any one of the following conditions: a. respiratory distress, respiratory rate ≥ 30/min; b. oxygen saturation of finger ≤ 93% in resting condition; c. arterial partial pressure of oxygen (PaO2) /oxygen concentration (FiO2) ≤ 300 mmHg (1 mmHg = 0.133 kPa); (4) critical type: patients meeting any one of the following criteria: a. respiratory failure requiring mechanical ventilation; b. shock; c. concomitant failure of other organs and requirement for intensive care unit (ICU) monitoring and treatment. In our study, the severity of disease was classified into two categories, non-severe type and severe type. Non-severe type includes mild-type, common-type or both, and SpO_2_≥90%. Otherwise, severe-type, critical-type or both and SpO_2_<90% are defined as severe type.

2. The composite endpoint was admission to an intensive care unit (ICU), the use of mechanical ventilation, or death [3].

3. Cardiac abnormality was defined by any one of following the course of disease: (1) complain of palpitation or chest distress; (2) TNT-HSST serum levels > 99th percentile upper reference limit (>28 pg/ml), the serum levels of troponin I (TNI) were above the 99th percentile of the upper reference limit (> 0.03 ug/L) using the Access AccuTnI+3 test, or increase in the levels of any of the other abovementioned cardiac markers; (3) NT-proBNP≥88.64 pg/mL is also characterized as a sign of Cardiac abnormality[4]; (4) new abnormalities on electrocardiography including sinus tachycardia [5-7].

4. Disease progression included death, progression from non-severe to severe, or severe-type to critical-type [8].

**References**

1. Metlay JP, Waterer GW, Long AC, et al. Diagnosis and Treatment of Adults with Community-acquired Pneumonia. An Official Clinical Practice Guideline of the American Thoracic Society and Infectious Diseases Society of America. Am J Respir Crit Care Med **2019**; 200(7): e45-e67.

2. Commission HC. The New Coronavirus Pneumonia Prevention and Control Guidelines of China. Available at: <http://www.nhc.gov.cn/>.

3. Guan WJ, Ni ZY, Hu Y, et al. Clinical Characteristics of Coronavirus Disease 2019 in China. N Engl J Med **2020**.

4. Gao L, Jiang D, Wen X, Cheng X, Sun M, al e. Prognostic value of NT-proBNP in patients with severe COVID-19. medRxiv 2020.

5. Liu R, Ming X, Xu O, Zhou J, Peng H, al e. Association of Cardiovascular Manifestations with In-hospital Outcomes in Patients with COVID-19: A Hospital Staff Data. medRxiv 2020.

6. Liu Y, Li J, Liu D, Song H, Chen C, al e. Clinical features and outcomes of 2019 novel coronavirus–infected patients with cardiac injury. medRxiv 2020.

7. Xu H, Hou K, Xu H, Li Z, Chen H, al e. Acute Myocardial Injury of Patients with Coronavirus Disease 2019. medRxiv 2020.

8. Hu L, Chen S, Fu Y, Gao Z, Long H, al e. Risk Factors Associated with Clinical Outcomes in 323 COVID-19 Patients in Wuhan, China. medRxiv 2020.

# Table S1 Characteristics of the included studies.

| **Rank** | **First author** | **Country** | **Year** | **Date of recruitment** | **Reason of non-eligible of quantitative synthesis** | **Province/city** | **Hospital** | **PMID\DOI** | **Total number of cases** | **Endpoints** | **NOS quality score** |
| --- | --- | --- | --- | --- | --- | --- | --- | --- | --- | --- | --- |
| 1 | Kaicai Liu | China | 2020 | 2020.01.21-2020.02.03 |  | Anhui | Six hospitals in Anhui province | 32193037 | 73 | severity | 5 |
| 2 | Jingyuan Liu | China | 2020 | 2020.01.13-2020.01.31 |  | Beijing | Beijing Ditan Hospital | 10.1101/2020.02.10.20021584 | 61 | severity | 5 |
| 3 | Sijia Tian | China | 2020 | 2020.01.20-2020.02.10 |  | Beijing | Beijing Emergency Medical Service | 32112886 | 262 | severity | 5 |
| 4 | Hui Hui | China | 2020 | 2020.01.21-2020.02.03 | duplicated patients | Beijing | Beijing Youan Hospital | 10.1101/2020.02.24.20027052 | 41 | severity | 5 |
| 5 | wen zhao | China | 2020 | 2020.01.21-2020.02.08 |  | Beijing | Beijing Youan Hospital | 10.1101/2020.03.13.20035436. | 77 | severity, hospitalization duration >14 days | 5 |
| 6 | Xu Chen | China | 2020 | 2020.01.23-2020.02.14 |  | Changsha, Loudi | first Hospital of Changsha and Loudi Central Hospital | 10.1101/2020.03.03.20030353 | 291 | severity | 5 |
| 7 | Zhichao Feng | China | 2020 | 2020.01.17-2020.02.01 |  | Changsha | Third Xiangya Hospital, Changsha Public Health Treatment Center, and Second People’s Hospital of Hunan | 10.1101/2020.02.19.20025296 | 141 | progression | 6 |
| 8 | Huayuan Xu | China | 2020 | 2020.01.02-2020.02.14 |  | Chengdu | West China Second University Hospital | 10.1101/2020.03.05.20031591. | 53 | cardiac abnormality | 6 |
| 9 | Lei Liu | China | 2020 | 2020.01.20-2020.02.03 |  | Chognqing | Chongqing Three Gorges Central Hospital | 10.1101/2020.02.20.20025536 | 51 | severity | 6 |
| 10 | Suxin Wan | China | 2020 | 2020.01.26-2020.02.04 | duplicated patients | Chognqing | Chongqing Three Gorges Central Hospital | 10.1101/2020.02.10.20021832 | 123 | severity | 5 |
| 11 | Di Qi | China | 2020 | 2020.01.19-2020.02.16 |  | Chognqing | Qianjiang central hospital of Chongqing, Chongqing three gorges central hospital and Chongqing public health medical center | 10.1101/2020.03.01.20029397 | 267 | severity | 5 |
| 12 | Kunhua Li | China | 2020 | 2020.01-2020.02 |  | Chognqing | the Second Affiliated Hospital of Chongqing Medical University | 32164090 | 83 | severity | 5 |
| 13 | Zhifeng Xu | China | 2020 | 2020.01.20-2020.02.06 |  | Foshan | the First people's hospital of Foshan | 10.1101/2020.03.03.20030775 | 21 | severity | 5 |
| 14 | Youbin Liu | China | 2020 | 2020.01.10-2020.02.24 |  | Guangzhou | Guangzhou Eighth People’s Hospital | 10.1101/2020.03.11.20030957 | 291 | cardiac abnormality | 6 |
| 15 | Yonghao Xu | China | 2020 | 2020.01.14-2020.02.20 |  | Guangzhou | the First Affiliated Hospital of Guangzhou Medical University, Dongguan People’s Hospital, etc | 10.1101/2020.03.03.20030668. | 45 | Invasive ventilation | 7 |
| 16 | Shijiao Yan | China | 2020 | 2020.01.22-2020.03.14 |  | Hainan | the Second Affiliated Hospital of Hainan Medical University | 10.1101/2020.03.19.20038539 | 168 | severity | 5 |
| 17 | Xiaowei Fang | China | 2020 | 2020.01.22-2020.02.18 |  | Hefei | Anhui Provincial Hospital |  | 79 | severity | 5 |
| 18 | Rong Qu | China | 2020 | 2020.01-2020.02 |  | Huizhou | Huizhou municipal central hospital | 32181903 | 30 | severity | 6 |
| 19 | Tian Gu | China | 2020 | 2019.12.18-2020.03.08 |  | mainland China |  | 10.1101/2020.03.23.20041848 | 321 | death | 6 |
| 20 | Yishan Zheng | China | 2020 |  |  | Nanjing | the Second Hospital of Nanjing | 10.1101/2020.02.19.20024885 | 88 | severity | 5 |
| 21 | Hongzhou Lu | China | 2020 | beofe 2020.02.07 |  | Shanghai | Shanghai CDC | 10.1101/2020.02.19.20025031 | 265 | severity | 5 |
| 22 | Min Cao | China | 2020 | 2020.01.20-2020.02.15 |  | Shanghai | Shanghai Public Health Clinical Centre | 10.1101/2020.03.04.20030395. | 198 | ICU | 6 |
| 23 | Ying Wen | China | 2020 | 2020.01.01-2020.02.28 |  | Shenzhen | Shenzhen Center of Disease Control and Prevention | 10.1101/2020.03.22.20035246 | 417 | severity | 5 |
| 24 | Qingxian Cai | China | 2020 | 2020.01.11-2020.02.06 |  | Shenzhen | the Third People's Hospital of Shenzhen | 10.1101/2020.02.17.20024018 | 298 | severity | 5 |
| 25 | Sakiko Tabata | Japan | 2020 | 2020.02.11-2020.02.25 |  | Tokyo | Self-Defense Ofces Central Hospital | 10.1101/2020.03.18.20038125 | 104 | severity | 5 |
| 26 | Bo Zhou | China | 2020 | 2020.02.05-2020.02.13 | different grouping methods of disease severity | Wuhan | West District of Union Hospital of Tongji Medical College | 32209382 | 34 | severe vs very severe | 5 |
| 27 | Jiatao Lu | China | 2020 | 2020.01.21-2020.02.05 |  | Wuhan | Wuhan Hankou Hospital | 10.1101/2020.02.20.20025510 | 577 | severity | 5 |
| 29 | Min Liu | China | 2020 | 2020.01.10-2020.01.31 |  | Wuhan | Affiliated hospital of Jianghan University | 32164090 | 30 | severity | 5 |
| 30 | Wei liu | China | 2020 | 2019.12.20-2020.01.15 |  | Wuhan | three tertiary hospitals in Wuhan | 32118640 | 78 | Progression | 6 |
| 31 | Mingli Yuan | China | 2020 | 2020.01.01-2020.01.25 |  | Wuhan | Central Hospital of Wuhan | 32191764 | 27 | death | 7 |
| 32 | Yafei Wang | China | 2020 | 2020.01.01-2020.02.10 |  | Wuhan | Central Hospital of Wuhan | 10.1101/2020.03.02.20029306 | 110 | severity | 5 |
| 33 | Ying Zhou | China | 2020 | 2020.01.01-2020.02.28 |  | Wuhan | Central Hospital of Wuhan | 10.1101/2020.03.24.20042119 | 377 | severity | 5 |
| 34 | Yanli Liu | China | 2020 | 2020.01.02-2020.02.01 |  | Wuhan | Central Hospital of Wuhan | 10.1101/2020.02.17.20024166. | 109 | ARDS | 7 |
| 35 | Ru Liu | China | 2020 | 2020.01.15-2020.01.24 |  | Wuhan | Central Hospital of Wuhan | 10.1101/2020.02.29.20029348 | 41 | cardiac abnormality | 6 |
| 36 | Chaolin Huang | China | 2020 | 2019.12.16-2020.01.02 |  | Wuhan | Jinyintan Hospital | 31986264 | 41 | ICU | 6 |
| 37 | Xiaobo Yang | China | 2020 | 2019.12.24-2020.01.26 |  | Wuhan | Jinyintan Hospital | 32105632 | 52 | death | 7 |
| 38 | Chaomin Wu | China | 2020 | 2019.12.25-2020.01.26 |  | Wuhan | Jinyintan Hospital | 32167524 | 201 | ARDS, death in ARDS | 7 |
| 39 | Fei Zhou | China | 2020 | 2020.12.29-2020.01.31 |  | Wuhan | Jinyintan Hospital and Wuhan Pulmonary Hospital | 32171076 | 191 | death | 6 |
| 40 | Jinjin Zhang | China | 2020 | 2020.01.16-2020.02.03 |  | Wuhan | No.7 hospital of Wuhan | 32077115 | 140 | severity | 5 |
| 41 | Qian Shi | China | 2020 | before 2020.02.15 | unique endpoint | Wuhan | Renmin Hospital of Wuhan University | 10.1101/2020.03.04.20031039 | 101 | survival ≤3d | 5 |
| 42 | Luwen Wang | China | 2020 | 2020.01.14-2020.02.13 |  | Wuhan | Renmin Hospital of Wuhan University | 10.1101/2020.02.19.20025288. | 116 | severity and ARDS | 5 |
| 43 | Yi Han | China | 2020 | 2020.02.01-2020.02.18 |  | Wuhan | Renmin Hospital of Wuhan University | 10.1101/2020.03.24.20040162 | 47 | severity | 5 |
| 44 | Ling Hu | China | 2020 | 2020.01.08-2020.02.20 |  | Wuhan | Tianyou Hospital | 10.1101/2020.03.25.20037721 | 323 | severity, unfavorable | 7 |
| 28 | Chen Chen | China | 2020 | 2020.01-2020.02 | different grouping methods of disease severity | Wuhan | Tongji Hospital | 32141280 | 150 | severity (critical vs non-critical) | 5 |
| 45 | Guang Chen | China | 2020 | 2019.12.19-2020.01.27 |  | Wuhan | Tongji hospital | 10.1101/2020.02.16.20023903. | 21 | severity | 5 |
| 46 | Tao Chen | China | 2020 | 2020.01.13-2020.02.12 |  | Wuhan | Tongji Hospital | 32217556 | 274 | death | 7 |
| 47 | Zhihua Wang | China | 2020 | 2020.02.23-2020.03.11 |  | Wuhan | Tongji hospital | 10.1101/2020.03.22.20041285 | 116 | death | 6 |
| 48 | Chuan Qin | China | 2020 | 2020.01.10-2020.02.12 |  | Wuhan | Tongji Hospital | 32161940 | 452 | severity | 5 |
| 49 | Lin Fu | China | 2020 | 2020.01.01-2020.01.30 |  | Wuhan | Union Hospital of Huazhong University of Science and Technology | 10.1101/2020.03.13.20035329. | 200 | death | 6 |
| 50 | Jing Liu | China | 2020 | 2020.01.05-2020.01.24 |  | Wuhan | Union Hospital of Huazhong University of Science and Technology | 10.1101/2020.02.16.20023671 | 40 | severity | 5 |
| 51 | Ling Mao | China | 2020 | 2020.01.16-2020.02.19 |  | Wuhan | Union Hospital of Huazhong University of Science and Technology | 10.1101/2020.02.22.20026500. | 214 | severity | 5 |
| 52 | Zhongliang Wang | China | 2021 | 2020.01.16-2020.01.29 |  | Wuhan | Union Hospital of Huazhong University of Science and Technology | 32176772 | 69 | Spo2<90% | 5 |
| 53 | Yudong Peng | China | 2020 | 2020.01.20-2020.02.15 |  | Wuhan | Union Hospital of Huazhong University of Science and Technology | 32120458 | 112 | severity,death | 6 |
| 54 | Fan Zhang | China | 2020 | 2019.12.25-2020.02.15 |  | Wuhan | Wuhan No.1 Hospital | 10.1101/2020.03.21.20040121 | 48 | non-surviver | 6 |
| 55 | Jianmin Jin | China | 2020 | 2020.01.29-2020.02.15 |  | Wuhan | Wuhan Union Hospital by the medical team of Beijing Tongren Hospital | 10.1101/2020.02.23.20026864 | 1056 | death | 6 |
| 56 | Dawei Wang | China | 2020 | 2020.01.01-2020.01.28 |  | Wuhan | Zhongnan Hospital of Wuhan University | 32031570 | 138 | ICU | 6 |
| 57 | Pingzheng Mo | China | 2020 | 2020.01.01-2020.02.05 | unique endpoint | Wuhan | Zhongnan Hospital of Wuhan University | 32173725 | 155 | Refractory | 6 |
| 58 | Guqin Zhang | China | 2020 | 2020.01.02-2020.02.10 |  | Wuhan | Zhongnan Hospital of Wuhan University | 10.1101/2020.03.02.20030452. | 221 | severity | 5 |
| 59 | Yao Na | China | 2020 | 2020.01.21-2020.02.21 | unique endpoint | Xi'an | Tangdu Hospital | 32153170 | 40 | liver injury | 6 |
| 60 | Weiliang Cao | China | 2020 | 2020.01.01-2020.02.16 |  | Xiangyang | the Xiangyang No.1 Hospital | 10.1101/2020.02.23.20026963 | 128 | severity | 5 |
| 61 | Jian Wu | China | 2020 | 2020.01.20-2020.02.19 |  | Yancheng | First People’s Hospital of Yancheng City, the Second People’s Hospital of Fuyang City, the Second People’s Hospital of Yancheng City, and the Fifth People’s Hospital of Wuxi | 32220033 | 280 | severity | 5 |
| 62 | Xiaowei Xu | China | 2020 | 2020.01.10-2020.01.26 | unique endpoint | Zhejiang | seven designated tertiary hospitals in Zhejiang province | 32075786 | 62 | Time since symptom onset ＞10 days | 5 |
| 63 | Bingwen Eugene FAN | Singapore | 2020 | 2020.01.23-2020.02.28 |  | Singapore | National Centre of Infectious Diseases of Singapore | 32129508 | 67 | ICU | 6 |
| 64 | Zhen Li | China | 2020 | 2020.01.06-2020.02.21 |  | Multiple cities | Wuhan Tongji hospital, Wuhan Pulmonary Hospital, Huangshi Central Hospital and Chongqing Southwest hospital | 10.1101/2020.02.08.20021212. | 193 | severity | 5 |
| 65 | Yang Xu | China | 2020 | 2020.02.07-2020.02.28 |  | Multiple cities | Zhongnan Hospital of Wuhan University, Chinese PLA General Hospital, Peking Union Medical College Hospital, and affiliated hospitals of Shanghai University of Medicine & Health Sciences. | 10.1101/2020.03.08.20031658 | 69 | severity | 5 |
| 66 | Weijie guan | China | 2020 | 2019.12.11-2020.01.31 |  | Multiple cities | Wuhan Jinyintan hospital, Union Hospital Affiliated to Tongji Medical College of Huazhong University of science and technology, Wuhan Central Hospital, Wuhan first hospital, Chengdu Public Health Clinical Medical Center | 32217650 | 1590 | severity, composite endpoint, death, ICU, invasive ventilation | 7 |
| 67 | Weijie guan | China | 2020 | 2019.12.11-2020.01.29 |  | Multiple cities |  | 32109013 | 1099 | severity, composite endpoint | 7 |
| 68 | Lei Gao | China | 2020 |  |  | Wuhan | Hubei General Hospital | 10.1101/2020.03.07.20031575 | 54 | cardiac abnormality | 7 |
| 69 | Huoshenshan (unpublished) | China | 2020 | 2020.02.03-2020.03.05 |  | Wuhan | Huoshenshan Hospital |  | 1780 | severity, composite endpoint, death, ICU, Invasive ventilation, ARDS | 7 |

# Table S2 Quantitative data synthesis for the associations of the epidemiological, comorbidity factors with prognosis of COVID-19

| **Variables** | **No of studies** | **Total cases** | **P _heterogeneity_** | **I^2^ (%)** | **RR (95% CIs)** | **P value** | **P _Egger_** |
| --- | --- | --- | --- | --- | --- | --- | --- |
| **Death** |  |  |  |  |  |  |  |
| Sex, male | 10 | 4214 | 0.443 | 0.0 | 1.23 (1.14-1.33) | <0.001 | 0.276 |
| Smoking | 4 | 2445 | 0.246 | 27.7 | 1.15 (0.84-1.57) | 0.395 | 0.061 |
| Current smoking | 2 | 2054 | 0.344 | 0.0 | 1.31 (0.64-2.67) | 0.459 | - |
| Ex-smoking | 2 | 2054 | 0.318 | 0.0 | 0.87 (0.26-2.95) | 0.826 | - |
| Contact with confirmed or suspect cases | 2 | 2054 | <0.001 | 98.8 | 1.11 (0.07-16.85) | 0.942 | - |
| Huanan seafood market exposure | 2 | 2054 | 0.918 | 0.0 | 5.84 (0.91-37.57) | 0.063 | - |
| Comorbidities | 8 | 4499 | <0.001 | 88.7 | 1.68 (1.32-2.13) | <0.001 | 0.248 |
| Hypertension | 11 | 4860 | <0.001 | 84.4 | 1.74 (1.31-2.30) | <0.001 | 0.418 |
| Diabetes | 10 | 4748 | 0.001 | 67.1 | 1.75 (1.27-2.41) | 0.001 | 0.057 |
| Malignancy | 6 | 3978 | 0.262 | 22.8 | 3.09 (1.59-6.00) | 0.001 | **0.006** |
| Cardiovascular disease | 11 | 4860 | <0.001 | 75.9 | 2.67 (1.60-4.43) | <0.001 | 0.654 |
| Coronary heart disease | 5 | 2452 | <0.001 | 87.7 | 3.16 (1.45-6.91) | 0.004 | 0.435 |
| Cerebrovascular disease | 6 | 3771 | 0.457 | 0.0 | 4.61 (2.51-8.47) | <0.001 | 0.766 |
| COPD | 4 | 3677 | 0.279 | 22.0 | 5.31 (2.63-10.71) | <0.001 | 0.107 |
| Respiratory system disease | 7 | 4472 | 0.185 | 31.8 | 3.22 (2.12-4.90) | <0.001 | 0.761 |
| Chronic kidney disease | 5 | 2219 | 0.477 | 0.0 | 7.10 (3.14-16.02) | <0.001 | 0.772 |
| Hepatitis B infection | 2 | 1864 | 0.973 | 0.0 | 1.18 (0.43-3.20) | 0.752 | - |
| Autoimmune disease | 2 | 1864 | 0.576 | 0.0 | 2.04 (0.27-15.58) | 0.491 | - |
|  |  |  |  |  |  |  |  |
| **Admission to ICU** |  |  |  |  |  |  |  |
| Sex, male | 5 | 2224 | 0.011 | 69.6 | 1.29 (1.13-1.47) | <0.001 | 0.651 |
| Smoking | 3 | 2019 | 0.742 | 0.0 | 0.85 (0.40-1.79) | 0.669 | 0.437 |
| Drinking | 2 | 1978 | 0.638 | 0.0 | 0.51 (0.10-2.55) | 0.411 | - |
| Huanan seafood market exposure | 3 | 1959 | 0.281 | 21.2 | 1.08 (0.44-2.69) | 0.863 | **0.037** |
| Comorbidities | 5 | 3747 | 0.038 | 60.5 | 1.82 (1.45-2.29) | <0.001 | 0.646 |
| Hypertension | 5 | 3747 | 0.601 | 0.0 | 2.31 (1.97-2.70) | <0.001 | 0.312 |
| Diabetes | 5 | 3747 | 0.084 | 51.4 | 1.88 (1.10-3.23) | 0.021 | 0.457 |
| Malignancy | 5 | 3747 | 0.427 | 0.0 | 2.52 (1.38-5.59) | 0.003 | 0.158 |
| Cardiovascular disease | 5 | 3747 | 0.511 | 0.0 | 2.74 (1.92-3.92) | <0.001 | 0.692 |
| Cerebrovascular disease | 3 | 3508 | 0.349 | 4.9 | 5.12 (2.86-9.17) | <0.001 | 0.273 |
| COPD | 4 | 3549 | 0.800 | 0.0 | 5.61 (2.68-11.76) | <0.001 | 0.740 |
| Respiratory system disease | 4 | 3549 | 0.613 | 0.0 | 4.66 (2.59-8.40) | <0.001 | 0.637 |
| Chronic kidney disease | 2 | 1728 | 0.344 | 0.0 | 1.37 (0.36-5.15) | 0.644 | - |
| Chronic liver disease | 3 | 377 | 0.906 | 0.0 | 0.50 (0.09-2.68) | 0.416 | 0.816 |
|  |  |  |  |  |  |  |  |
| **Composite endpoint** |  |  |  |  |  |  |  |
| Sex, male | 2 | 2879 | 0.001 | 91.3 | 1.48 (0.95-2.29) | 0.082 | - |
| Smoking | 2 | 2879 | 0.604 | 0.0% | 2.67(1.91-3.73) | <0.001 | - |
| Current smoking | 2 | 2879 | 0.038 | 76.7 | 1.59 (0.64-3.98) | 0.322 | - |
| Ex-smoking | 2 | 2879 | 0.035 | 77.4 | 2.34 (0.24-22.93) | 0.466 | - |
| Contact with confirmed or suspect cases | 2 | 2879 | 0.392 | 0.0 | 1.02 (0.84-1.24) | 0.827 | - |
| Comorbidities | 2 | 3370 | <0.001 | 95.3 | 1.96 (1.06-3.60) | 0.031 | - |
| Hypertension | 2 | 3370 | 0.011 | 84.5 | 2.20 (1.44-3.36) | <0.001 | - |
| Diabetes | 2 | 3370 | 0.002 | 89.2 | 2.20 (0.86-5.66) | 0.101 | - |
| Malignancy | 2 | 3370 | 0.072 | 69.1 | 3.76 (1.00-14.16) | 0.051 | - |
| Cardiovascular disease | 2 | 3370 | 0.927 | 0.0 | 3.09 (2.09-4.57) | <0.001 | - |
| Coronary heart disease | 2 | 3370 | 0.473 | 0.0 | 3.36 (2.15-5.25) | <0.001 | - |
| Cerebrovascular disease | 2 | 3370 | 0.225 | 32.0 | 4.10 (2.34-7.18) | <0.001 | - |
| COPD | 2 | 3370 | 0.185 | 43.0 | 8.52 (4.36-16.65) | <0.001 | - |
| Respiratory system disease | 2 | 3370 | 0.185 | 43.0 | 8.52 (4.36-16.65) | <0.001 | - |
|  |  |  |  |  |  |  |  |
| **ARDS** |  |  |  |  |  |  |  |
| Sex, male | 3 | 2090 | 0.464 | 0.0 | 1.15 (1.01-1.30) | 0.033 | 0.353 |
| Hypertension | 3 | 2090 | 0.377 | 0.0 | 1.90 (1.57-2.30) | <0.001 | 0.520 |
| Diabetes | 3 | 2090 | 0.068 | 62.9 | 3.07 (1.28-7.36) | 0.012 | 0.066 |
| Cardiovascular disease | 3 | 2090 | 0.244 | 29.2 | 2.26 (1.43-3.58) | <0.001 | 0.422 |
| Cerebrovascular disease | 2 | 1889 | 0.152 | 51.2 | 3.15 (1.23-8.04) | 0.016 | - |
| COPD | 2 | 1889 | 0.140 | 54.1 | 2.59 (0.94-7.17) | 0.066 | - |
| Respiratory system disease | 2 | 1889 | 0.303 | 5.6 | 2.44 (1.20-4.97) | 0.014 | - |
|  |  |  |  |  |  |  |  |
| **Invasive ventilation** |  |  |  |  |  |  |  |
| Sex, male | 2 | 1825 | 0.403 | 0.0 | 1.35 (1.11-1.64) | 0.002 | - |
| Smoking | 2 | 1825 | 0.657 | 0.0 | 0.94 (0.41-2.15) | 0.885 | - |
| Contact with confirmed or suspect cases | 2 | 1825 | 0.020 | 81.5 | 1.44 (0.77-2.71) | 0.253 | - |
| Family cluster | 2 | 1825 | 0.646 | 0.0 | 1.58 (1.13-2.14) | 0.006 | - |
| Comorbidities | 3 | 3415 | 0.005 | 81.2 | 1.83 (1.19-2.79) | 0.006 | 0.569 |
| Hypertension | 3 | 3415 | 0.131 | 50.9 | 2.35 (1.92-2.89) | <0.001 | 0.366 |
| Diabetes | 3 | 3415 | 0.131 | 50.8 | 1.85 (1.24-2.76) | 0.003 | **0.021** |
| Malignancy | 3 | 3415 | 0.397 | 0.0 | 1.79 (0.66-4.88) | 0.252 | 0.110 |
| Cardiovascular disease | 3 | 3415 | 0.844 | 0.0 | 2.90 (1.63-5.15) | <0.001 | 0.618 |
| Cerebrovascular disease | 2 | 3370 | 0.602 | 0.0 | 3.98 (1.77-8.93) | 0.001 | - |
| COPD | 2 | 3370 | 0.383 | 0.0 | 6.53 (2.70-15.84) | <0.001 | - |
| Respiratory system disease | 3 | 3415 | 0.260 | 25.7 | 4.34 (2.04-9.26) | <0.001 | 0.567 |
|  |  |  |  |  |  |  |  |
| **Cardiac abnormality** |  |  |  |  |  |  |  |
| Sex, male | 4 | 439 | 0.211 | 33.6 | 1.33 (1.02-1.72) | 0.036 | 0.624 |
| Smoking | 2 | 94 | 0.448 | 0.0 | 1.12 (0.33-3.73) | 0.860 | - |
| Exposure to Hubei Province | 2 | 344 | 0.464 | 0.0 | 1.18 (0.76-1.83) | 0.473 | - |
| Contact with confirmed or suspect cases | 2 | 94 | 0.408 | 0.0 | 0.94 (0.65-1.36) | 0.735 | - |
| Hypertension | 4 | 439 | 0.947 | 0.0 | 2.97 (1.65-5.34) | <0.001 | 0.610 |
| Diabetes | 4 | 439 | 0.695 | 0.0 | 1.85 (0.90-3.81) | 0.094 | 0.247 |
| Cardiovascular disease | 4 | 439 | 0.915 | 0.0 | 4.90 (1.82-13.21) | 0.002 | 0.177 |
| Coronary heart disease | 3 | 386 | 0.819 | 0.0 | 5.37 (1.74-16.54) | 0.003 | 0.408 |
| COPD | 3 | 148 | 0.881 | 0.0 | 2.30 (0.48-11.02) | 0.296 | 0.480 |
| Respiratory system disease | 3 | 148 | 0.881 | 0.0 | 2.30 (0.48-11.02) | 0.296 | 0.480 |
|  |  |  |  |  |  |  |  |
| **Disease progression** |  |  |  |  |  |  |  |
| Sex, male | 2 | 219 | 0.853 | 0.0 | 1.38 (0.93-2.05) | 0.106 | - |
| Smoking | 2 | 219 | 0.068 | 70.0 | 2.70 (0.14-51.96) | 0.511 | - |
| Hypertension | 2 | 219 | 0.547 | 0.0 | 2.90 (1.45-5.81) | 0.003 | - |
| Diabetes | 2 | 219 | 0.746 | 0.0 | 3.30 (1.08-10.07) | 0.036 | - |
| COPD | 2 | 219 | 0.848 | 0.0 | 7.48 (1.60-35.05) | 0.011 | - |
| Respiratory system disease | 2 | 219 | 0.848 | 0.0 | 7.48 (1.60-35.05) | 0.011 | - |

# Figure S1 Forest plot of association between sex and disease severity.

# Figure S2 Forest plot of association between smoking and disease severity.

# Figure S3 Forest plot of association between current smoker and disease severity.

# Figure S4 Forest plot of association between ex-smoker and disease severity.

# Figure S5 Forest plot of association between drinking and disease severity.

# Figure S6 Forest plot of association between local residents of Wuhan and disease severity.

# Figure S7 Forest plot of association between exposure history to Hubei province and disease severity.

# Figure S8 Forest plot of association between contact with confirmed or suspect cases and disease severity.

# Figure S9 Forest plot of association between family cluster and disease severity.

# Figure S10 Forest plot of association between Huanan seafood market exposure and disease severity.

# Figure S11 Forest plot of association between comorbidity and disease severity.

# Figure S12 Forest plot of association between hypertension and disease severity.

# Figure S13 Forest plot of association between diabetes and disease severity.

# Figure S14 Forest plot of association between malignancy and disease severity.

# Figure S15 Forest plot of association between cardiovascular disease and disease severity.

# Figure S16 Forest plot of association between coronary heart disease and disease severity.

# Figure S17 Forest plot of association between cerebrovascular disease and disease severity.

# Figure S18 Forest plot of association between cardiovascular/ cerebrovascular disease and disease severity.

# Figure S19 Forest plot of association between COPD and disease severity.

# Figure S20 Forest plot of association between respiratory system disease and disease severity.

# Figure S21 Forest plot of association between chronic kidney disease and disease severity.

# Figure S22 Forest plot of association between chronic liver disease and disease severity.

# Figure S23 Forest plot of association between hepatitis B infection and disease severity.

# Figure S24 Forest plot of association between lithiasis and disease severity.

# Figure S25 Forest plot of association between autoimmune disease and disease severity.

# Figure S26 Forest plot of association between abnormal lipid metabolism and disease severity.

# Figure S27 Forest plot of association between digestive disease and disease severity.

# Figure S28 Forest plot of association between thyroid disease and disease severity.

# Figure S29 Forest plot of association between tuberculosis and disease severity.

# Figure S30 Forest plot of association between nervous system disease and disease severity.

# Figure S31 Forest plot of association between endocrine system disease and disease severity.

# Figure S32 Forest plot of association between death and sex.

# Figure S33 Forest plot of association between death and smoking.

# Figure S34 Forest plot of association between death and current smoking.

# Figure S35 Forest plot of association between death and ex-smoking.

# Figure S36 Forest plot of association between death and contact with confirmed or suspect cases.

# Figure S37 Forest plot of association between death and Huanan seafood market exposure.

# Figure S38 Forest plot of association between death and comorbidities.

# Figure S39 Forest plot of association between death and hypertension.

# Figure S40 Forest plot of association between death and diabetes.

# Figure S41 Forest plot of association between death and malignancy.

# Figure S42 Forest plot of association between death and cardiovascular disease.

# Figure S43 Forest plot of association between death and coronary heart disease.

# Figure S44 Forest plot of association between death and cerebrovascular disease.

# Figure S45 Forest plot of association between death and COPD.

# Figure S46 Forest plot of association between death and respiratory system disease.

# Figure S47 Forest plot of association between death and chronic kidney disease.

# Figure S48 Forest plot of association between death and hepatitis B infection.

# Figure S49 Forest plot of association between death and autoimmune disease.

# Figure S50 Forest plot of association between admission to ICU and sex.

# Figure S51 Forest plot of association between admission to ICU and smoking.

# Figure S52 Forest plot of association between admission to ICU and drinking.

# Figure S53 Forest plot of association between admission to ICU and Huanan seafood market exposure.

# Figure S54 Forest plot of association between admission to ICU and comorbidities.

# Figure S55 Forest plot of association between admission to ICU and hypertension.

# Figure S56 Forest plot of association between admission to ICU and diabetes.

# Figure S57 Forest plot of association between admission to ICU and malignancy.

# Figure S58 Forest plot of association between admission to ICU and cardiovascular disease.

# Figure S59 Forest plot of association between admission to ICU and cerebrovascular disease.

# Figure S60 Forest plot of association between admission to ICU and COPD.

# Figure S61 Forest plot of association between admission to ICU and respiratory system disease.

# Figure S62 Forest plot of association between admission to ICU and chronic kidney disease.

# Figure S63 Forest plot of association between admission to ICU and chronic liver disease.

# Figure S64 Forest plot of association between composite endpoint and sex.

# Figure S65 Forest plot of association between composite endpoint and smoking.

# Figure S66 Forest plot of association between composite endpoint and current smoking.

# Figure S67 Forest plot of association between composite endpoint and ex-smoking.

# Figure S68 Forest plot of association between composite endpoint and contact with confirmed or suspect cases.

# Figure S69 Forest plot of association between composite endpoint and comorbidities.

# Figure S70 Forest plot of association between composite endpoint and hypertension.

# Figure S71 Forest plot of association between composite endpoint and diabetes.

# Figure S72 Forest plot of association between composite endpoint and malignancy.

# Figure S73 Forest plot of association between composite endpoint and cardiovascular disease.

# Figure S74 Forest plot of association between composite endpoint and coronary heart disease.

# Figure S75 Forest plot of association between composite endpoint and cerebrovascular disease.

# Figure S76 Forest plot of association between composite endpoint and COPD.

# Figure S77 Forest plot of association between composite endpoint and respiratory system disease.

# Figure S78 Forest plot of association between ARDS and sex.

# Figure S79 Forest plot of association between ARDS and hypertension.

# Figure S80 Forest plot of association between ARDS and diabetes.

# Figure S81 Forest plot of association between ARDS and cardiovascular disease.

# Figure S82 Forest plot of association between ARDS and cerebrovascular disease.

# Figure S83 Forest plot of association between ARDS and COPD.

# Figure S84 Forest plot of association between ARDS and respiratory system disease.

# Figure S85 Forest plot of association between invasive ventilation and sex.

# Figure S86 Forest plot of association between invasive ventilation and smoking.

# Figure S87 Forest plot of association between invasive ventilation and contact with confirmed or suspect cases.

# Figure S88 Forest plot of association between invasive ventilation and family cluster.

# Figure S89 Forest plot of association between invasive ventilation and comorbidities.

# Figure S90 Forest plot of association between invasive ventilation and hypertension.

# Figure S91 Forest plot of association between invasive ventilation and diabetes.

# Figure S92 Forest plot of association between invasive ventilation and malignancy.

# Figure S93 Forest plot of association between invasive ventilation and cardiovascular disease.

# Figure S94 Forest plot of association between invasive ventilation and cerebrovascular disease.

# Figure S95 Forest plot of association between invasive ventilation and COPD.

# Figure S96 Forest plot of association between invasive ventilation and respiratory system disease.

# Figure S97 Forest plot of association between cardiac abnormality and sex.

# Figure S98 Forest plot of association between cardiac abnormality and smoking.

# Figure S99 Forest plot of association between cardiac abnormality and exposure to Hubei Province.

# Figure S100 Forest plot of association between cardiac abnormality and contact with confirmed or suspect cases.

# Figure S101 Forest plot of association between cardiac abnormality and hypertension.

# Figure S102 Forest plot of association between cardiac abnormality and diabetes.

# Figure S103 Forest plot of association between cardiac abnormality and cardiovascular disease.

# Figure S104 Forest plot of association between cardiac abnormality and coronary heart disease.

# Figure S105 Forest plot of association between cardiac abnormality and COPD.

# Figure S106 Forest plot of association between cardiac abnormality and respiratory system disease.

# Figure S107 Forest plot of association between disease progression and sex.

# Figure S108 Forest plot of association between disease progression and smoking.

# Figure S109 Forest plot of association between disease progression and hypertension.

# Figure S110 Forest plot of association between disease progression and diabetes.

# Figure S111 Forest plot of association between disease progression and COPD.

# Figure S112 Forest plot of association between disease progression and respiratory system disease.

# Figure S113 Forest plot of association between age and severity.

# Figure S114 Forest plot of association between age and death.

# Figure S115 Forest plot of association between age and admission to ICU.

# Figure S116 Forest plot of association between age and composite endpoint.

# Figure S117 Forest plot of association between age and ARDS.

# Figure S118 Forest plot of association between age and invasive ventilation.

# Figure S119 Forest plot of association between age and cardiac abnormality.

# Figure S120 Forest plot of association between age and disease progression.
